# Therapeutic Investigation of Zingerone Against Pentylenetetrazole-Induced Kindled Seizures in Mice

**DOI:** 10.5812/ijpr-162878

**Published:** 2025-09-21

**Authors:** Xiaocui Tian, Xiaoke Xu

**Affiliations:** 1 Department of Rehabilitation Medicine, Xi'an Children' Hospital, Xi'an, China

**Keywords:** Cognition Deficits, Reduced Glutathione, Thiobarbituric Acid Reactive Substances, Tumor Necrosis Factor, Water T-maze, Water Y-maze

## Abstract

**Background:**

Epilepsy is a condition characterized by frequent bursts of neuro-electrical impulse activity in the brain, involving the expression of transient receptor potential vanilloid receptor 1 (TRPV1). Zingerone (ZO) is known to possess a multitude of regulatory mechanisms for TRP channels. However, clear evidence of ZO in the regulation of TRPV1 expression has not yet been reported.

**Objectives:**

The present study was designed to evaluate the therapeutic role of ZO against pentylenetetrazole (PTZ)-induced kindled seizures (KS) in mice.

**Methods:**

The KS were induced by intraperitoneal (i.p.) administration of three doses of PTZ (35 mg/kg/day) in mice on every alternate day (day 1, 3, and 5). Additionally, the PTZ challenge test was performed on day 20. The ZO doses of 25 and 50 mg/kg; TRPV1 antagonist, i.e., selective TRPV1 antagonist (SB-366791, 10 mg/kg); and a combination of ZO and SB-366791 were administered orally (p.o.). The seizure score was assessed using Racine's scoring system on day 20. Changes in KS-associated spatial cognition were assessed by the water Y-maze and water T-maze tests. The hippocampal tissue biomarkers, i.e., thiobarbituric acid reactive substances (TBARS), reduced glutathione (GSH), tumor necrosis factor-alpha (TNF-α), and TRPV1 expression were estimated.

**Results:**

The ZO attenuates the PTZ-induced changes in Racine's scores and spatial cognition effects in Y-maze and water T-maze tests. Furthermore, ZO also ameliorates the PTZ-induced biomarker changes.

**Conclusions:**

Hence, ZO possesses therapeutic potential against KS conditions in mice via regulation of TRPV1 channel functions. However, more extensive studies are required to prove this therapeutic potency in different seizure conditions across various animal species.

## 1. Background

Epilepsy is a condition characterized by frequent bursts of neuro-electrical impulse activity in the brain. The risk factors for epilepsy include brain trauma, autoimmune disease, and metabolic abnormalities, including chemicals like pentylenetetrazole (PTZ) (1). Transient receptor potential vanilloid receptor 1 (TRPV1) is a calcium-permeable channel that is highly expressed in the cornu ammonis area of the epileptic brain (2). Calcium channels are responsible for regulating neuronal impulses and promoting neuronal firing (excitation) in the brain (3). Medications for the management of seizures primarily regulate the neuronal membrane potential by blocking sodium channels and excitatory neurotransmitter functions (4). Neuronal calcium channel blockers possess a weak effect on reducing seizure frequency and can cause withdrawal side effects (5). However, the identification and exploration of newer neuronal receptor functions with molecular aspects of calcium channels are important for managing patients with severe epileptic attacks.

The activation of TRPV1 is involved in the progression of epilepsy. The TRPV1 is known to open the cation channel, which leads to epilepsy via the enhancement of neuronal excitability and neuroinflammation (6). From a pharmacological perspective, TRPV1 is a widely accepted target for epilepsy management. Moreover, endocannabinoids like anandamide activate TRPV1, contributing to the enhancement of pro-convulsant action (2). Furthermore, the administration of TRPV1 antagonists, such as capsazepine, attenuates the progression of epilepsy (7).

The calcium-permeable TRPV1 channels also contribute to neuronal firing and cause epilepsy (6). Zingerone (ZO) is a non-toxic methoxyphenolic compound with multiple biological and pharmacological effects (8). Some studies have revealed that ZO activates the TRPV1 channel via direct interaction with the TRPV1 channel pore (9). However, repeated administration of ZO is known to cause the desensitization of TRPV1 proteins. The ZO modulates neuronal TRP channels by sensitization and desensitization in dose- and duration-dependent manners (10). Nevertheless, there is currently no conclusive proof linking ZO to the control of TRPV1 expression in epileptic disorders.

## 2. Objectives

With a correlation to TRPV1 channel modulatory effects, the current work aims to assess the therapeutic role of ZO against PTZ-induced kindling seizures in mice.

## 3. Methods

### 3.1. Animals Used

In this investigation, male Swiss albino mice (12 months, 20 - 30 g) were employed. The animals were fed a conventional laboratory meal and had free access to water. The animals were kept in a central animal home with a 12-hour day/night cycle. The institutional animal ethics committee (IAEC permission no.: 20240119) approved this experimental design. The experiments followed the IAEC criteria.

### 3.2. Experimental Design

The experimental design included five groups, each consisting of eight mice. Group I animals acted as the naive control. Group II animals acted as negative controls. This group of rats was subjected to recurrent intraperitoneal (i.p.) treatment with a 35 mg/kg dose of PTZ to induce kindled seizures (KS) over three alternate days, i.e., day 1, 3, and 5. In KS-induced mice, groups III and IV received oral ZO (25 and 50 mg/kg, respectively) for 15 days after the third dose of PTZ (from day 6). Group V mice received the selective TRPV1 antagonist (SB-366791, 10 mg/kg; p.o.) for 15 days (from day 6) after the third dose of PTZ in KS-induced mice. Groups VI and VII received a combination of ZO (25 and 50 mg/kg; p.o.) with SB-366791 (10 mg/kg; p.o.), respectively, for 15 days after the third dose of PTZ administration. Additionally, the animals in groups II to VII were employed in the PTZ challenge test on day 20 by administering a 35 mg/kg dose of PTZ.

### 3.3. Induction and Assessment of Kindled Seizures

Three sub-convulsant dosages of PTZ (35 mg/kg) were repeatedly injected intraperitoneally on days 1, 3, and 5 to induce KS. According to Tambe et al. (11), with modification from Shimada and Yamagata (12), this method can induce kindling seizures in mice. Convulsive behaviors were monitored for 30 minutes after the PTZ challenge test on day 20. Van Erum et al.'s modified Racine's scoring system was employed to evaluate the severity of the Seizure Index (SI) on the twentieth day (13).

### 3.4. Assessment of Kindled Seizures-Induced Spatial Cognition by the Water Y-Maze Test

The KS-induced changes in spatial cognition were assessed by water Y-maze tests as described by the method of Deacon (14) with the modification of Kraeuter et al. (15). The water Y-maze test was used for the assessment of KS-associated spatial cognitive functions. The spatial cognitive function of mice was assessed by placing the mice at the starting point of the water Y-maze test device. An acclimatization period of 5 minutes was allotted before performing the test observation. During this acclimatization period, the animal was exposed to all three arms. Mice were placed at the starting point to assess spatial cognitive functions by determining transfer latency (TL). The cut-off time was maintained for two minutes for this cognitive functional observation.

### 3.5. Assessment of Kindled Seizures-Induced Spatial Cognition by Water T-Maze Test

Water T-maze tests were used to investigate the effects of KS on spatial cognition, following the method reported by Guariglia and Chadman (16) and Locchi et al. (17). The water T-maze test assessed spatial cognitive abilities linked with KS. The spatial cognitive function of mice was assessed by placing the mice at the starting point of the water T-maze test device. An acclimatization period of 5 minutes was allotted before performing the test observation. During this acclimatization period, the animal was exposed to all three arms. If the animal did not reach the red and green chambers, guidance was given to reach the corner of each arm. The next day, mice were placed at the starting point to assess spatial cognitive functions by determining TL. The cut-off time was maintained for two minutes for this cognitive functional observation.

### 3.6. Estimation of Hippocampal Tissue Markers Changes

Following the evaluation of behavioral data, mice were sacrificed by cervical dislocation. A phosphate buffer with a pH of 7.4 that had been chilled with ice was used to homogenize the hippocampus tissue. Tissue biomarkers, such as thiobarbituric acid reactive substances (TBARS), reduced glutathione (GSH), tumor necrosis factor-alpha (TNF-α), and TRPV1 expression levels, were evaluated using the aliquot.

#### 3.6.1. Estimation of Thiobarbituric Acid Reactive Substances

The TBARS estimation is an indicator of lipid peroxidation, which is produced during cellular stress and damaged conditions. The Ohkawa et al. (18) approach was used to assess the tissue TBARS levels. In simple terms, 0.2 mL of the aliquot was combined with 1.5 mL of 30% acetic acid, 0.2 mL of sodium dodecyl sulfate (8.1%), and 1.5 mL of thiobarbituric acid (0.8%). Distilled water was used to create the entire volume of 4 mL. A spectrophotometer (DU 640B Spectrophotometer, Beckman Coulter Inc., Brea, CA, USA) set to 532 nanometers was used to quantify the variations in the pink-colored chromogen.

#### 3.6.2. Estimation of Reduced Glutathione

The GSH estimation is an indicator of oxidative stress, which is depleted during cellular stress and damaged conditions. Ellman's method for estimating tissue GSH levels was reported (19). In summary, 10% w/v trichloroacetic acid (1:1 ratio) was combined with the tissue supernatant. These mixtures were centrifuged at 123 G force for ten minutes at 4°C. Two milliliters of disodium hydrogen phosphate (0.3 M) were combined with roughly 0.5 milliliters of a clear aliquot. A freshly made 5,5-Dithiobis (2-Nitro Benzoic Acid and DTNB; 0.001 M) solution was then added in an amount of approximately 0.25 mL. Using a spectrophotometer (DU 640B Spectrophotometer, Beckman Coulter Inc., Brea, CA, USA) set to 412 nanometers, the variations in the yellow-colored chromogen were measured.

#### 3.6.3. Estimation of Tumor Necrosis Factor-alpha and Transient Receptor Potential Vanilloid Receptor 1 Expression

The estimation of TNF-α and TRPV1 is an indicator of inflammation and activation of TRPV1 channels, respectively, which are expressed during KS conditions with neuronal damage. It was calculated using the guidelines provided by the commercial enzyme-linked immunosorbent assay (ELISA) kit (MyBioSource, Selangor Darul Ehsan, Malaysia). The changes in chromogen were measured by a microplate reader (BioTek Microplate Instruments, Penang, Malaysia) at 450 nanometer wavelengths.

#### 3.6.4. Estimation of Tissue Total Proteins

The approach published by Lowry et al. (20) was used to estimate the total proteins in the tissue. In summary, 5 mL of Lowry's reagents, 1 mL of phosphate buffer, and roughly 0.15 mL of an aliquot were combined in test tubes. The Folin-Ciocalteu reagent (0.5 mL) was then added and quickly vortexed. A spectrophotometer (DU 640B Spectrophotometer, Beckman Coulter Inc., Brea, CA, USA) set to 750 nanometers was used to measure the variations in the purple-colored chromogen.

### 3.7. Statistical Analysis

The standard deviations (SD) of each data set were displayed. GraphPad Prism software version 5.0, developed by Dotmatics (R&D scientific software firm, San Diego, CA, USA), was used to statistically evaluate the data from the KS score, water Y-maze, and water T-maze tests using the two-way analysis of variance (ANOVA) test and the Bonferroni post-hoc test. Additionally, one-way ANOVA and Tukey's multiple range tests were used to assess the results on TBARS, GSH, TNF-α, TRPV1, and total protein levels. A statistically significant probability (P) value was defined as one that was less than 0.05.

## 4. Results

### 4.1. Effect of Zingerone on the Changes of Kindled Seizures-Induced Racine's Score

**Figure 1.**
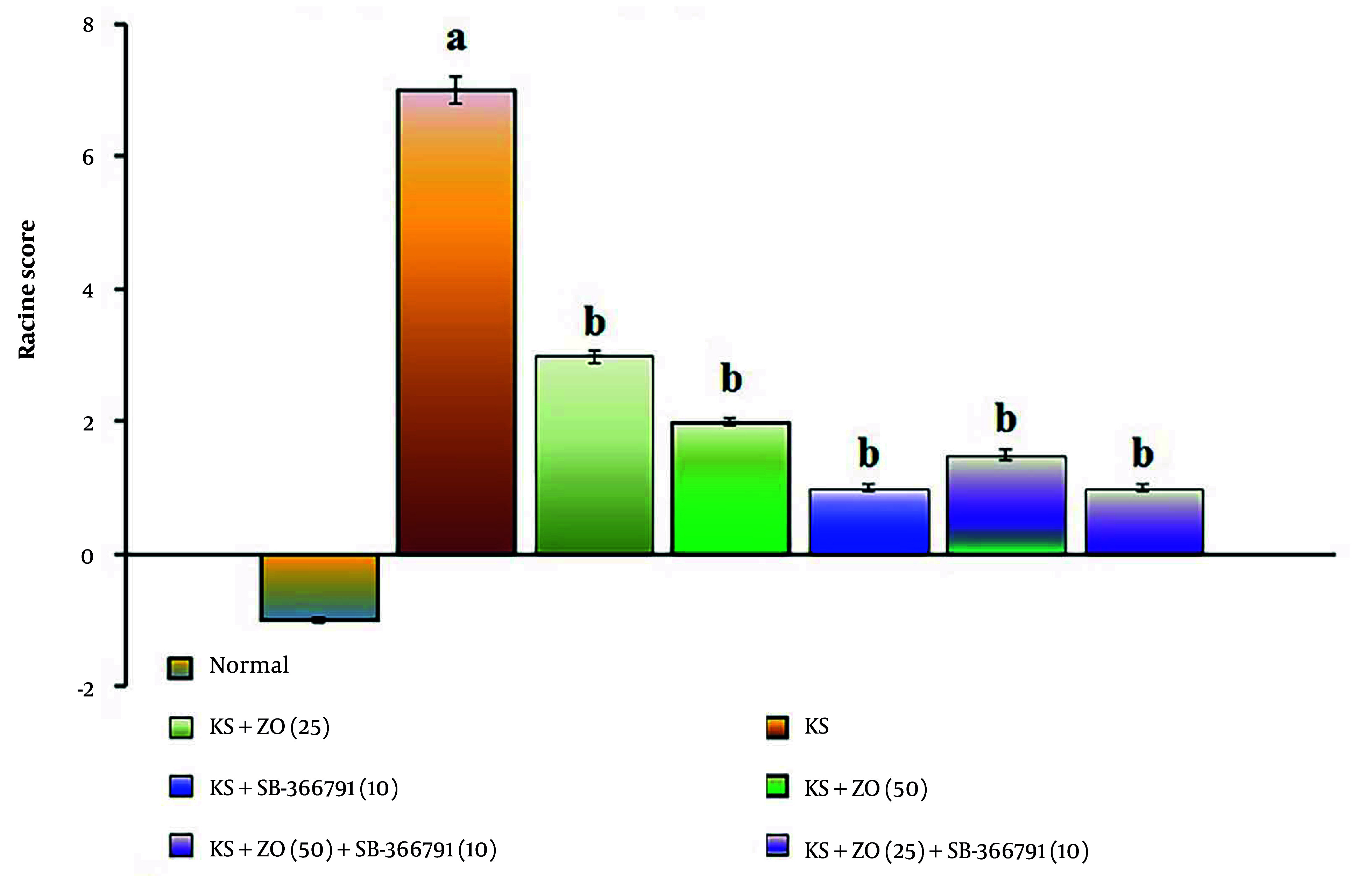
Effect of ZO on the changes of the KS-induced Racine's score. The numbers in parentheses represent a dose of mg/kg. The results are presented as the mean SD, with n = 8 mice per group. a, P < 0.05 versus the control group; and b, P < 0.05 versus the KS group. (Abbreviations: KS, kindled seizure; SB-366791, selective TRPV1 antagonist; and ZO, zingerone).

The administration of PTZ (35 mg/kg; i.p., for three alternate days, i.e., day 1, 3, and 5) resulted in a significant increase in Racine's score compared to the normal group (P < 0.01). The data showed similar results in the day 20 PTZ challenge test, suggesting that PTZ produces KS due to alterations in neuronal membrane potential (excitation). When compared to the KS group, the oral administration of ZO (25 and 50 mg/kg; for 15 days) and in combination with SB-366791 decreased the KS-induced Racine's score in a dose-dependent manner. The SB-366791 (10 mg/kg; p.o.) alone treatment group experienced a similar effect. The ZO has the same ameliorative potential against KS via TRPV1 antagonist action, indicated by a reduction of KS-induced Racine's scores. The data results are displayed in Figure 1. 

### 4.2. Effect of Zingerone on the Changes of Kindled Seizures-Induced Spatial Cognition in the Water Y-Maze Test

**Figure 2.**
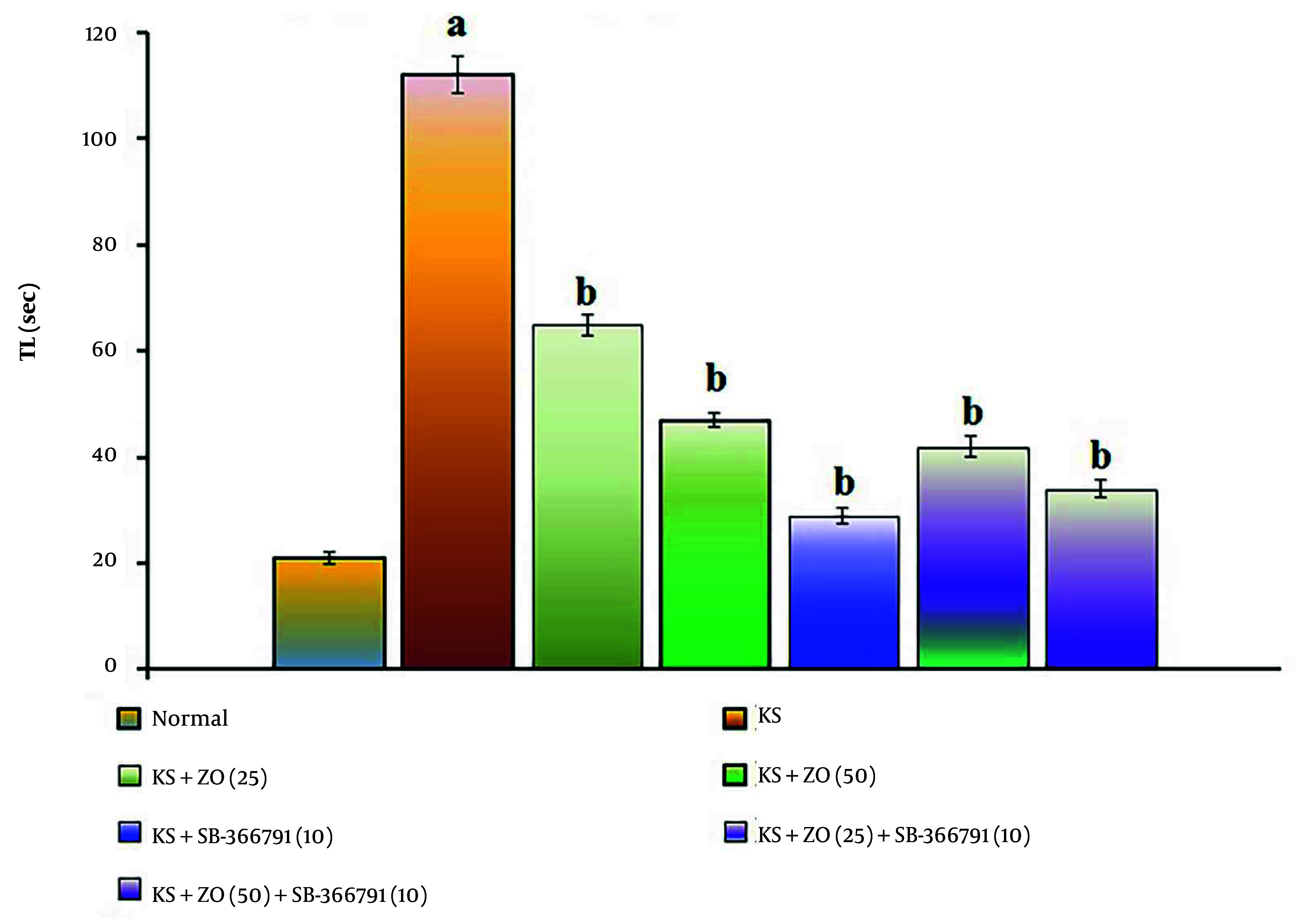
Effect of ZO on the changes of the KS-induced spatial cognition in the water Y-maze test. The numbers in parentheses represent a dose of mg/kg. The results are presented as the mean SD, with n = 8 mice per group. a, P < 0.05 versus the control group; and b, P < 0.05 versus the KS group. (Abbreviations: KS, kindled seizure; SB-366791, Selective TRPV1 antagonist; TL, transfer latency; and ZO, zingerone).

The administration of PTZ (35 mg/kg; i.p., for three alternate days, i.e., day 1, 3, and 5) resulted in a significant increase in TL duration (P < 0.05). This suggests that PTZ impairs spatial cognition in KS-induced mice relative to the normal group due to alterations in neuronal excitation brought on by neuronal injury and neurotransmitter dysfunction compared to the KS group (P < 0.037). The PTZ-induced increase in TL length is attenuated in a dose-dependent manner by oral ZO treatment (25 and 50 mg/kg) and in combination with SB-366791 for 15 consecutive days. The SB-366791 (10 mg/kg; p.o.) alone treatment group experienced a similar effect. The decrease in TL length suggests that ZO can alleviate KS, which is comparable to TRPV1 antagonist therapies. The data results are displayed in Figure 2. 

### 4.3. Effect of Zingerone on the Changes of Kindled Seizures-Induced Spatial Cognition in the Water T-Maze Test

**Figure 3.**
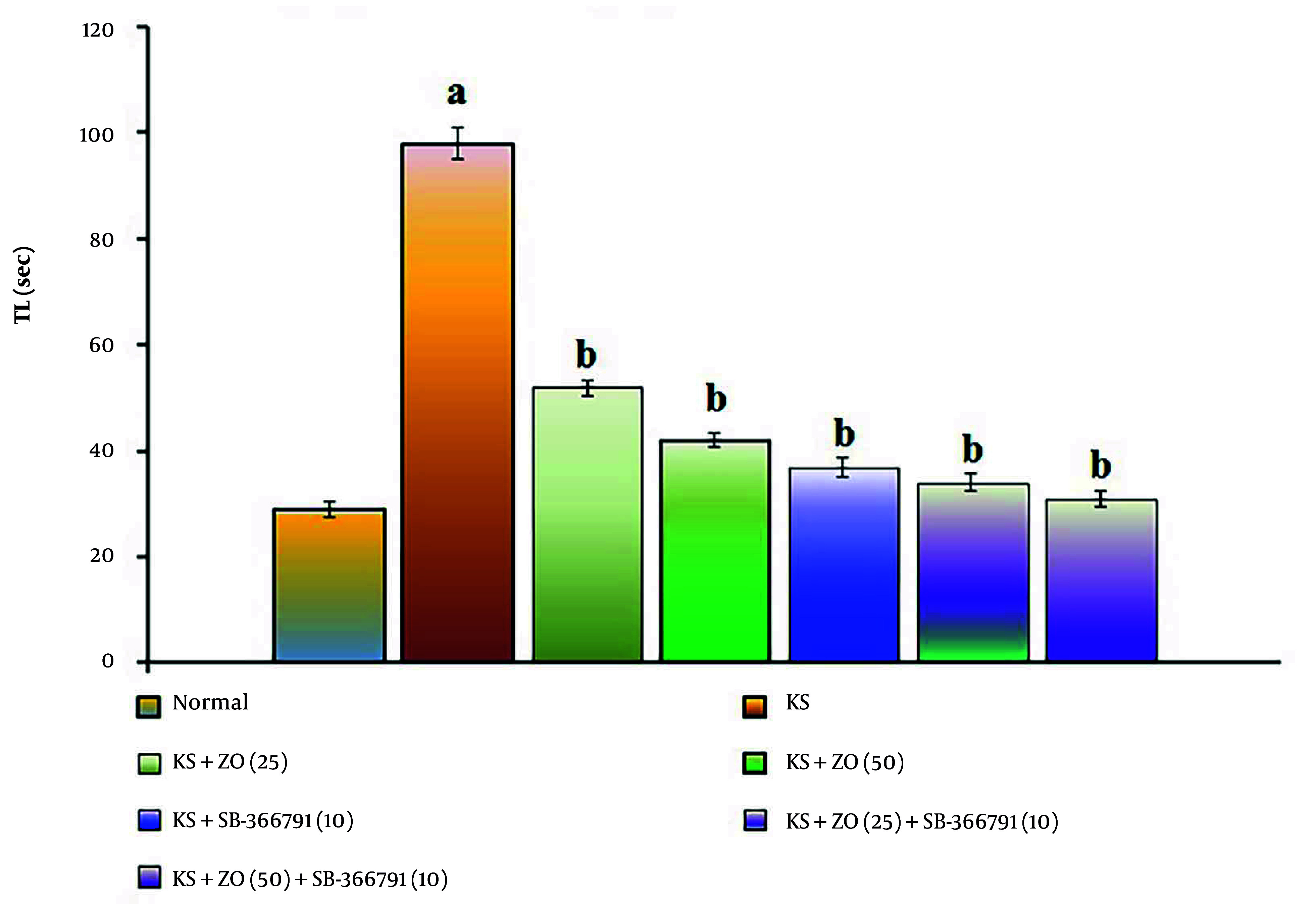
Effect of ZO on the changes of the KS-induced spatial cognition in the water T-maze test. The numbers in parentheses represent a dose of mg/kg. The results are presented as the mean SD, with n = 8 mice per group. a, P < 0.05 versus the control group; b, P < 0.05 versus the KS group. (Abbreviations: KS, kindled seizure; SB-366791, Selective TRPV1 antagonist; TL, transfer latency; and ZO, zingerone).

The administration of PTZ (35 mg/kg; i.p., for three alternate days, i.e., day 1, 3, and 5) resulted in a significant increase in TL duration compared to the normal group (P < 0.001). This indicates that PTZ impairs spatial cognition in KS-induced mice due to alterations in neuronal excitation linked to neurotransmitter dysfunction and neuronal death. In comparison to the KS group, the PTZ-induced increase in TL length was reduced in a dose-dependent manner by oral administration of ZO (25 and 50 mg/kg) and in combination with SB-366791 for 15 days. The SB-366791 (10 mg/kg; p.o.) alone treatment group experienced a similar effect. The decrease in TL length suggests that ZO can alleviate KS, which is comparable to TRPV1 antagonist therapies. The data results are displayed in Figure 3. 

### 4.4. Effect of Zingerone on the Changes in Hippocampal Tissue Markers

The administration of PTZ (35 mg/kg; i.p., for three alternate days, i.e., day 1, 3, and 5) resulted in a significant rise in TBARS content, expression of TNF-α and TRPV1 proteins, and reduced GSH content levels. This indicates that PTZ causes neuronal damage via oxidative stress, lipid peroxidation, and alteration of neuronal membrane potentials when compared to the normal group (P < 0.029). Compared to the KS group, the PTZ-induced changes in the aforementioned hippocampus tissue markers were lessened when ZO (25 and 50 mg/kg) was administered orally, either alone or in combination with SB-366791, for 15 consecutive days. The SB-366791 (10 mg/kg; p.o.) alone treatment group experienced a similar effect. The regulation of tissue biomarkers and neuronal calcium ion channel proteins indicates that ZO possesses ameliorative potential against KS, similar to TRPV1 antagonist treatments. The results of the data are tabulated in Table 1. 

**Table 1.** Effect of Zingerone on the Changes of Hippocampal Tissue Markers ^a,^ ^b^

Groups (mg/kg) ^c^	TBARS (nmol/mg of Protein)	GSH (µmol/mg of Protein)	TNF-α (pg/mg of Protein)	TRPV1 (pg/mg of Protein)
**Normal**	1.21 ± 0.04	54.82 ± 1.6	0.32 ± 0.02	41.34 ± 1.4
**KS (35)**	4.37 ± 0.05 ^d^	10.89 ± 1.3 ^d^	4.63 ± 0.07 ^d^	113.71 ± 2.1^d^
**KS+ZO (25)**	3.16 ± 0.06 ^e^	32.47 ± 1.7 ^e^	1.58 ± 0.05 ^e^	56.14 ± 1.8 ^e^
**KS+ZO (50)**	2.53 ± 0.04 ^e^	42.83 ± 1.5 ^e^	1.12 ± 0.06 ^e^	51.46 ± 1.9 ^e^
**KS+SB-366791 (10)**	1.48 ± 0.06 ^e^	49.93 ± 1.1 ^e^	0.71 ± 0.04 ^e^	44.89 ± 1.7 ^e^
**KS+ZO (25)+SB-366791 (10)**	2.93 ± 0.04 ^e^	37.19 ± 2.2 ^e^	1.16 ± 0.03 ^e^	49.72 ± 1.4 ^e^
**KS+ZO (50)+SB-366791 (10)**	1.78 ± 0.05 ^e^	47.14 ± 0.9 ^e^	0.98 ± 0.06 ^e^	47.92 ± 1.1 ^e^

Abbreviations: TBARS, thiobarbituric acid reactive substances; GSH, glutathione; TNF-α, tumor necrosis factor-alpha; TRPV1, transient receptor potential vanilloid receptor 1; KS, kindled seizure; ZO, zingerone; SB-366791, selective TRPV1 antagonist.

^a^ Values are expressed as mean ± standard deviations (SD).

^b^ N = 8 mice per group.

^c^ The numbers in parentheses represent a dose of mg/kg.

^d^ P < 0.042 versus the control group.

^e^ P < 0.039 versus the KS group.

## 5. Discussion

The i.p. administration of PTZ (35 mg/kg; i.p., for three alternate days, i.e., day 1, 3, and 5) in mice demonstrated a significant development of the KS Racine score in the PTZ challenge test. It also causes cognitive dysfunction due to the alteration of neuronal membrane calcium ion channel proteins, expression of inflammatory cytokines, and induction of oxidative stress. The PTZ was causing the KS and behavioral alterations in mice (21). This study's findings show that PTZ demonstrated changes in spatial memory in the water Y-maze and water T-maze tests comparable to normal animals. Additionally, PTZ decreased the amount of GSH in the hippocampus tissue of mice and increased the levels of TBARS, TNF-α, and TRPV1. These findings are comparable to those of other studies (22). Our results indicate that KS conditions cause severe neuronal oxidative stress and neuroinflammation in mice with the expression of TRPV1 channel proteins. Furthermore, it causes kindling seizures in mice; hence, it is known as the chemical kindling model of epilepsy (23). Long-term exposure to PTZ also induces the release of neurotransmitter precursors, i.e., phenylalanine and isoleucine, for glutamate synthesis (24). The present studies also revealed that PTZ causes the reduction of neuronal endogenous antioxidant molecules, i.e., GSH. Furthermore, PTZ also enhances the MDA products, which are released due to neuronal membrane lipid peroxidation via modulation of the TRPM2 channel, i.e., transient receptor potential melastatin-2 (25). Besides, PTZ also causes KS in rats via the expression of central TRPV1 receptors (26).

Natural products, such as ZO, are known to activate the TRPV1 receptor (27). Furthermore, another study evidenced that the vanilloid group of ZO is not sufficient to activate the TRPV1 channels (28). The synthetic analog of the TRPV1 agonist, capsaicin, activates the TRPV1 channels at the initial stage, whereas later it acts as an antagonist of the TRPV1 channel (29). Hence, natural pungent compounds like ZO have pleiotropic actions on the same receptor based on the exposure episodes and duration in experimental animals (30). In epileptic conditions, ZO possesses hippocampal neuroprotection against lithium chloride and pilocarpine-induced neuroinflammation and neurodegeneration (31).

Furthermore, the aging process is known to decline cognitive functions. According to the Jackson Aging Center, a 12-month-old mouse is considered a mature adult or middle-aged mouse (32). This may be one of the limitations of this study. However, after 12 months, mice show a decline in cognitive functions and physical abilities (32, 33). Hence, a 12-month-old mouse was used in this study. The ZO has been reported to possess potential anti-inflammatory and antioxidant actions in maximal electroshock and PTZ-induced seizures in mice via regulation of superoxide dismutase, catalase, and TBARS (34). Furthermore, ZO also reduced hippocampal neurodegeneration with the regulation of nuclear factor-kappa B pathways mediated by TNF-α in lithium chloride and pilocarpine-induced epilepsy in male mice (31).

Mechanistically, compounds of *Zingiber officinale*, like ZO, directly interact with the S4 - S5 linker of the TRPV1 cation channel and regulate nociception (27). Furthermore, the activation of TRPV1 possesses both pro-inflammatory and anti-inflammatory actions depending upon the expression of TRPV1 in specific cells. It also plays a key role in the modulation of immune reactions, leading to antioxidant and anti-inflammatory actions (35). The present study also shows that ZO possesses potential anti-convulsant action against PTZ-induced KS in mice due to its antioxidant, anti-inflammatory, and TRPV1 receptor modulatory actions.

The modulation of TRPV1 plays a key role in the pathogenesis of epilepsy via neuroinflammatory reactions in brain tissue. Furthermore, the administration of anti-inflammatory drugs, such as acetaminophen, and TRPV1 antagonists, such as capsazepine, attenuates the epilepsy condition in mice (7). The present results showed that ZO, in combination with a TRPV1 antagonist (SB-366791; 10 mg/kg), exerts anticonvulsant action with anti-inflammatory effects (reduction of TBARS and TNF-α levels) compared to SB-366791 treatment alone. The primary pathogenesis of PTZ-induced kindling seizure is due to the modulation of the TRPV1 channel and the gamma-aminobutyric acid – A receptor antagonistic action, leading to hyper-excitation of neuronal tissue (36).

Moreover, the effect of ZO with the modulation of the TRPV1 channel in different pathological conditions, such as microglia activation and glutamate receptors associated with various types of KS conditions, still needs to be investigated in experimental animal models (37). Animal models of KS have been established by the application of corneal and hippocampal electrical stimuli, optogenetics (light) exposure, and picrotoxin and kainic acid administration in mice and rats (38-40). Even chemical methods of KS have been studied in zebrafish species (41). These kinds of KS models can be used to investigate the effect of ZO on KS to establish its effects on the modulation of TRPV1 channels. Furthermore, this research hypothesis will be extended in our next research work.

### 5.1. Conclusions

The oral administration of ZO and a SB-366791, alone and in combination, ameliorates PTZ-induced KS in mice by reducing TBARS, TNF-α, and TRPV1 levels and raising GSH levels. The anticonvulsant action of ZO is due to its potential antioxidant, anti-lipid peroxidation, anti-inflammatory, and TRPV1 channel modulatory actions. However, a more extensive study is required in various animal models of KS to establish the ameliorative actions of ZO on KS with modulation of TRPV1 channel functions.

## Data Availability

The dataset used and analyzed in the current study is available upon request.
